# Transcriptional Mediators Kto and Skd Are Involved in the Regulation of the IMD Pathway and Anti-*Plasmodium* Defense in *Anopheles gambiae*

**DOI:** 10.1371/journal.pone.0045580

**Published:** 2012-09-25

**Authors:** Yang Chen, Yuemei Dong, Simone Sandiford, George Dimopoulos

**Affiliations:** W. Harry Feinstone Department of Molecular Microbiology and Immunology, Bloomberg School of Public Health, Johns Hopkins University, Baltimore, Maryland, United States of America; Centro de Pesquisas René Rachou, Brazil

## Abstract

The malarial parasite *Plasmodium* must complete a complex lifecycle in its *Anopheles* mosquito host, the main vector for *Plasmodium*. The mosquito resists infection with the human malarial parasite *P. falciparum* by engaging the NF-κB immune signaling pathway, IMD. Here we show that the conserved transcriptional mediators Kto and Skd are involved in the regulation of the mosquito IMD pathway. RNAi-mediated depletion of Kto and Skd in the *Anopheles gambiae* cell line L5-3 resulted in a decrease in the transcript abundance of *Cec1,* which is controlled by the IMD pathway. Silencing the two genes also resulted in an increased susceptibility of the mosquito to bacterial and *Plasmodium falciparum* infection, but not to infection with the rodent malaria parasite *P. berghei*. We also showed that Kto and Skd are not transcriptional co-activators of Rel2 or other key factors of the IMD pathway; however, they participate in the regulation of the IMD pathway, which is crucial for the mosquito’s defense against *P. falciparum*.

## Introduction

Malaria, one of the deadliest diseases in the world, is responsible for the deaths of over one million people annually. *Anopheles* mosquitoes are the main vectors for protozoan parasites of the genus *Plasmodium*, which cause the disease. About 24 h after the female mosquito ingests a blood meal from an infected mammalian host, the parasites develop into ookinetes that invade the epithelium of the mosquito midgut. The innate immune system of the mosquito is the main defense against the *Plasmodium* parasites [1], [2], [3], [4]. Therefore, a better understanding of the interaction between the parasite and the mosquito’s immune system could facilitate the development of novel disease control and prevention strategies. Recent studies have shown that the IMD pathway is the most important immune pathway in the mosquito’s defense against the human pathogen *P. falciparum* [5], [6]. Several anti-*Plasmodium* immune effectors controlled by the IMD pathway, such as TEP1, APL1, LRRD7 and FBN9, have also been characterized with regard to their anti-parasitic activity [3], [7], [8], [9], [10], [11], [12], [13].

Kohtalo (Kto) and Skuld (Skd), also known as Med12 and Med13, or TRAP230 and TRAP240, are two major transcriptional mediator proteins [14], [15], [16], [17], [18], [19], [20], [21], [22], [23], [24], [25]. These two transcriptional mediators are part of a group of evolutionally conserved proteins that act as transcriptional co-activators, forming complexes that bridge regulatory regions to the RNA polymerase II initiation complex [26], [27], [28], [29], [30]. Studies in *Drosophila*, zebrafish, and *Caenorhabditis elegans* have shown that Kto and Skd are required for several specific developmental processes [14], [15], [16], [17], [18], [19], [20], [21], [22], [23], [24], [25].

Previous studies of Kto and Skd in flies have focused on their function in the wing and eye disks [14], [15]. Kto- and Skd-mutant cells proliferate, survive, and initiate but do not complete differentiation; most notably, these cells do not respect compartment boundaries, leading to a disorganized tissue architecture [14], [15]. Kto and Skd have been shown to be essential for the function of the transcription factor Atonal (Ato) in the spatial patterning of proneural clusters in the morphogenetic furrow [25]. In *C. elegans*, Kto is an essential gene [20], [24] and is required for asymmetric cell division in the T blast cell lineage [18]. In zebrafish, mutation of Kto results in abnormal development of the brain, neural crest, and kidney [17].

Here we show that Kto and Skd regulate the *A. gambiae* IMD immune pathway. Silencing of Kto or Skd in a mosquito cell line resulted in a decrease in the transcript abundance *of Cec1*, which is known to be controlled by the IMD pathway. Kto and Skd silencing *in vivo* increased the mosquitoes’ susceptibility to infection with *Staphylococcus aureus* and *Escherichia coli*, as well as with *P. falciparum*, but not *P. berghei*. Our study shows, for the first time, that the transcriptional mediators Kto and Skd are involved in the regulation of the IMD immune signaling pathway.

## Results

### Kto Regulates the IMD Pathway

**Figure 1:**
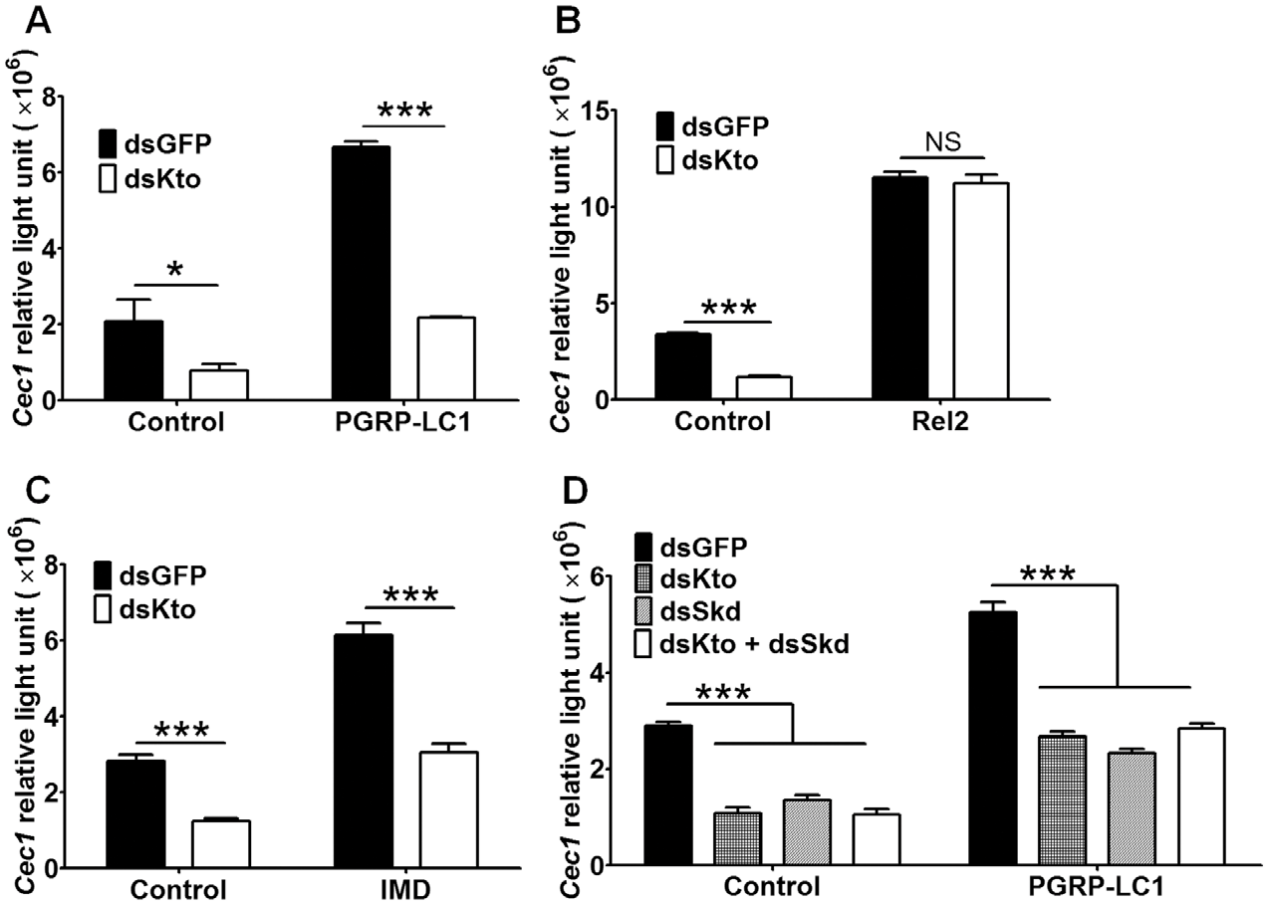
Kto and Skd regulate the Imd pathway in the L3-5 cell line. A) Silencing of *Kto* impairs the IMD pathway, and this impairment cannot be rescued by over-expression of PGRP-LC1; B) over-expression of Rel2 abolished the effect of silencing *Kto*; C) over-expression of IMD did not rescue the phenotype caused by silencing *Kto*; D) silencing *Kto* and *Skd* resulted in similar phenotypes. Shown are representative results from one of three independent repeats with similar trends. *, *p*<0.05; ***, *p*<0.001.

In order to determine the role of Kto in the regulation of the IMD pathway, we monitored the activation of the immune pathway after using RNAi to silence the expression of Kto in the L3-5 mosquito cell line [31], which expresses firefly luciferase under a IMD pathway-regulated *Cec1* promoter. We used *Renilla* luciferase under the control of the *Drosophia Actin5c* promoter as an internal control [32]. This RNAi treatment resulted in a prominent down-regulation of the *Cec1*-driven luciferase gene (as measured by relative light units), indicating a down-regulation of the IMD pathway (Fig. 1A).

We then wanted to investigate which components of the IMD pathway are regulated by Kto (i.e., at which level of the IMD pathway Kto exerts its regulatory activity). Since Kto functions as a transcriptional co-activator in other organisms, we hypothesized that it may serve as a transcriptional co-activator of Rel2, the transcription factor of the IMD pathway. To test this possibility, we knocked down Kto in L3-5 cells that over-expressed the active form of Rel2; this form of Rel2 lacks the inhibitory domain and therefore can be translocated into the nucleus and directly activate gene expression [11]. Over-expression of the activated form of Rel2 strongly activated the IMD pathway, as has been reported previously ([11]; Fig. 1B, black bars). However, silencing of Kto had no measurable effect on the *Cec1* promoter activity in the Rel2 over-expressing cells (Fig. 1B), suggesting that Kto may not function as a co-activator of Rel2 but rather plays a role upstream of this NF-κB transcription factor, although it is possible that over-expression of Rel2 in the cells could have masked the effect of Kto silencing to some degree. We have previously shown that over-expression of the IMD pathway pattern recognition receptor PGRP-LC1 increases the expression of *Cec1* [33], [34]. Silencing of Kto in PGRP-LC1 over-expressing cells impaired this IMD pathway activation, as measured by *Cec1* expression (Fig. 1A), suggesting that Kto acts downstream of PGRP-LC1.

To further investigate which part of the IMD pathway is targeted by Kto, we silenced it in IMD-over-expressing L3-5 cells; IMD acts downstream of PGRP-LC1 and upstream of Rel2. In response to Kto silencing, we saw impairment in the boosting effect of over-expressing IMD (Fig. 1C). These results suggest that Kto targets one or several components of the IMD pathway that act downstream of PGRP-LC1/IMD and upstream of Rel2.

### Skd Regulates the IMD Pathway

Since previous studies conducted in *Drosophila* have shown that Kto acts together with Skd, and that Kto and Skd mutants have similar phenotypes in abnormally developing eyes [25], we wanted to investigate whether Skd acts together with Kto in the regulation of the IMD immune signaling pathway. For this purpose, we silenced Kto and Skd separately and together in the L3-5 cell line and measured *Cec1* expression by the luciferase assay. The results indicated that single and double silencing had similar effects on the IMD pathway (Fig. 1D). Silencing Kto and Skd separately showed a similar degree of *Cec1* activity repression with or without PGRP-LC1 over-expression, and simultaneous silencing of both genes did not further decrease the *Cec1* activity. Our results and those previous studies in other species, indicate that Kto and Skd are likely to act together on the same target factor, or alternatively, on different targets of the IMD pathway.

### Kto and Skd do not Regulate the Transcription of IMD Pathway Factors

**Figure 2:**
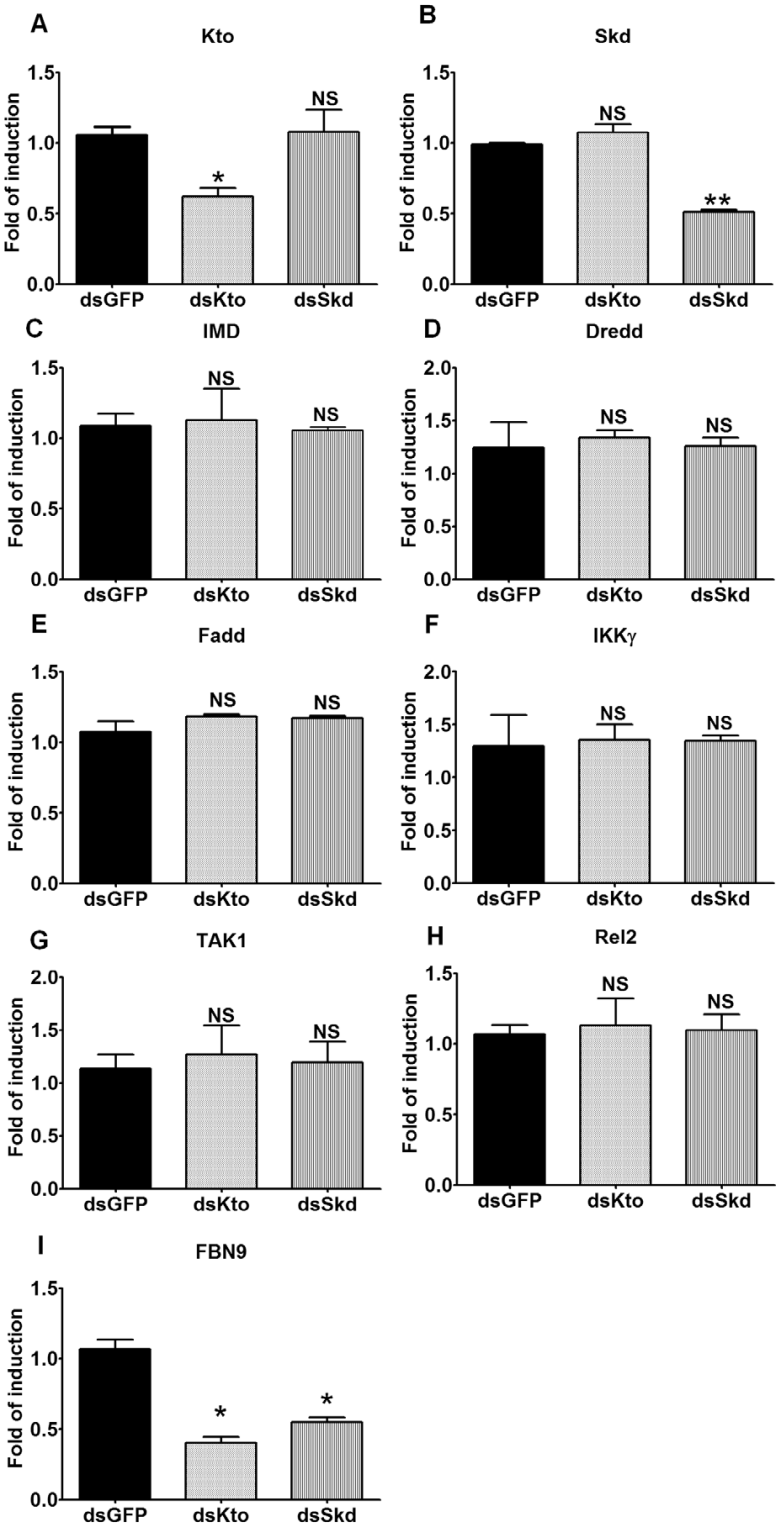
Kto and Skd do not transcriptionally regulate the transcript abundance of IMD pathway factors. The transcript abundance of A) Kto; B) Skd; C) IMD; D) Dredd; E) Fadd; F) IKKg; G) TAK1; H) Rel2; and I) Fbn9, after *Kto* and *Skd* silencing. Shown are representative results from one of three independent repeats with similar trends. *, *p*<0.05; **, *p*<0.01.

Kto and Skd are known to be involved in gene transcription, acting as co-activators of transcription factors. However, our results did not indicate that they are transcriptional co-activators of Rel2 (Fig. 1B). It was possible that they are instead involved in the transcription of IMD pathway factors, thereby influencing the pathway’s activity. In order to investigate this possibility, we studied the effect of Kto and Skd silencing at 48 h after dsRNA treatment on the transcript abundance of five major IMD pathway factors that act downstream of PGRP-LC and upstream of Rel2. At a Kto and Skd silencing efficiency of approximately 50% (Fig. 2AB) the mRNA abundance of *Imd*, *Dredd*, *Fadd*, *Ikk-γ*,*Tak1* and *Rel2* was not altered from that of GFP dsRNA-treated control cells (Fig. 2C–H). We also tested whether *Kto* and *Skd* silencing had any effect on the transcript abundance of the anti-*Plasmodium* immune effector *Fbn9*, which is controlled by the IMD pathway [8]. Indeed the expression of *Fbn9* was down-regulated in both *Kto* and *Skd* silenced groups (Fig. 2I).

### Kto and Skd Influence Mosquitoes’ Resistance to Bacterial Challenges

**Figure 3:**
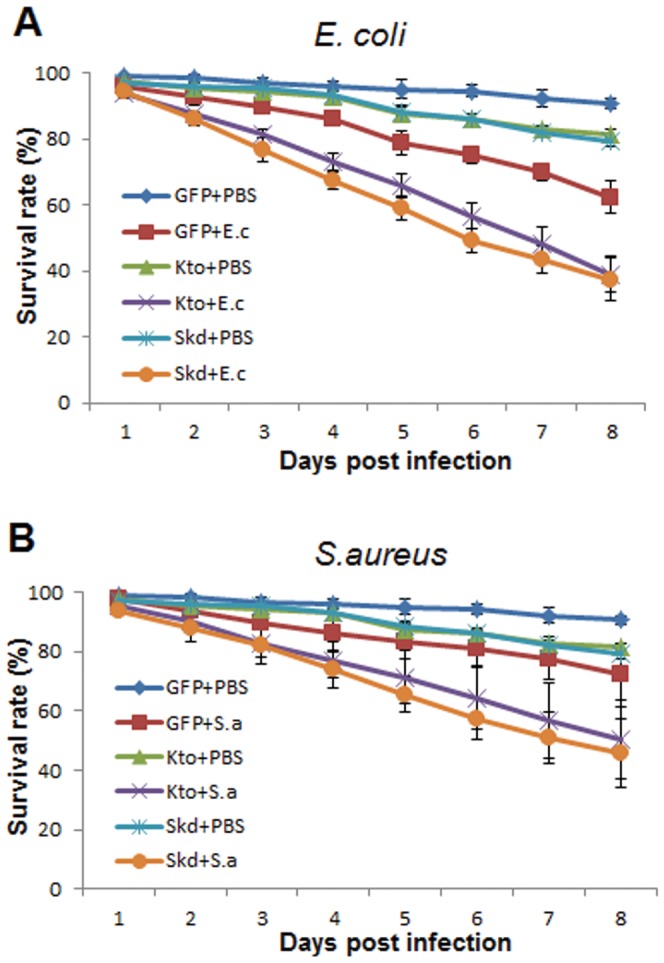
Kto and Skd influence A. gambiae resistance to bacterial challenge. A) Survival rates of GFP dsRNA-, Kto dsRNA- and Skd dsRNA-injected mosquitoes after *E. coli* infection; B) survival rates of GFP dsRNA-, Kto dsRNA- and Skd dsRNA-injected mosquitoes after *S. aureus* infection. PBS was injected into control mosquito cohorts. The effect of gene silencing on the mortality of mosquitoes after bacterial infection, as compared to GFP dsRNA-treated controls, was determined by Kaplan-Meier survival analysis; p-values are listed in Tables S1 and S2. The mean survival percentage for all three biological replicates are shown, together with the standard errors.

Previous studies have shown that the IMD pathway mediates the mosquitoes’ defense against infections with both Gram-positive and Gram-negative bacteria. For example, transgenic mosquitoes over-expressing Rel2 show enhanced resistance to both Gram-positive and Gram-negative bacteria [11]. To investigate the potential role of Kto and Skd in the mosquitoes’ resistance to challenge with the Gram-positive bacterium *S. aureus* and the Gram-negative bacterium *E. coli* DH5α, we silenced Kto and Skd prior to injection of the mosquitoes with live bacteria, and then monitored survival rates for 8 days. Silencing of either Kto or Skd impaired the mosquitoes’ capacity to defend against both *E. coli* and *S. aureus* (Fig. 3, Table S1 and S2), thereby indicating a role for these factors in the anti-bacterial defense, most likely mediated through the IMD pathway.

### Kto and Skd Influence Mosquitoes’ Resistance to *P. falciparum* and *P. berghei* Infection by Affecting the IMD Pathway

**Figure 4:**
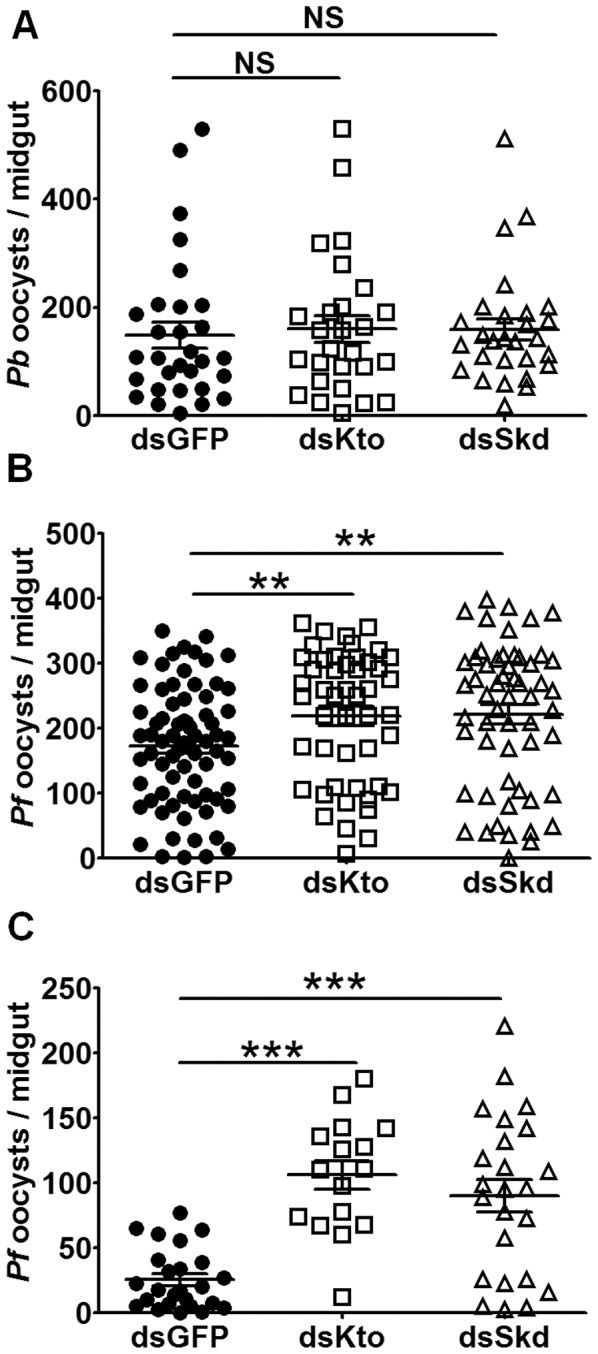
Kto and Skd regulate mosquito susceptibility to *P. falciparum* but not *P. berghei* infection. *Anopheles gambiae* were injected with GFP, Kto or Skd dsRNA. Three days later, they were fed on either a *P. falciparum* gametocyte culture or a *P. berghei-* infected mouse. A) *P. berghei* oocyst loads of antibiotic-treated mosquitoes; B) *P. falciparum* oocyst loads of antibiotic-treated mosquitoes; C) *P. falciparum* oocycst loads of non-antibiotic-treated mosquitoes. Shown are representative results from one of 3 independent repeats with similar trends. **, *p*<0.01; ***, *p*<0.001.

We have previously shown that the *Anopheles* IMD pathway mediates resistance to infection with *P. falciparum* but not *P. berghei* [5]. To investigate whether Kto and Skd influence the mosquitoes’ susceptibility to infection with these parasite species, we performed gene-silencing experiments in conjunction with infection assays. Independent antibiotic-treated mosquito cohorts were injected with dsRNAs targeting each gene and were then fed on either a *P. falciparum* gametocyte culture or *P. berghei*-infected mouse 4 days later. At 8 days after feeding, the mosquito midguts were dissected, and infection intensity (as indicated by parasite oocyst number) was determined. Silencing either Kto or Skd resulted in an increased susceptibility to *P. falciparum* (Fig. 4B) but not *P. berghei* infection (Fig. 4A), when compared to the GFP dsRNA-treated controls. Antibiotic-treated mosquitoes were used because we had observed a rather high mortality of gene-silenced non-antibiotic-treated mosquitoes after feeding when the midgut microbiota proliferates to high numbers [35] (data not shown). The IMD pathway is known to be implicated in the control of the midgut microbiota and its impairment may thus have led to mortality caused by bacterial infection. (data not shown). However, the gene silencing-mediated resistance to *P. falciparum* infection was in the same range for both antibiotic-treated and non-treated mosquito cohorts (Fig. 4C). The resistance specificity of Kto or Skd gene-silenced mosquitoes to the two parasite species was therefore consistent with that seen for the Imd pathway.

## Discussion

Mediator complexes represent a group of evolutionally conserved proteins involved in transcriptional activation. Studies in *C. elegans*, zebrafish, and *Drosophila* have shown that Kto and Skd are involved in regulating several specific developmental processes. Here we have shown for the first time that Kto and Skd also play key roles in regulating the IMD pathway in the mosquito, an essential part of the innate immune system’s defense against bacterial and *P. falciparum* infections.

Although Kto and Skd are transcriptional co-activators, our results show that they are not likely to act as such for Rel2 (Fig. 1B), the transcription factor of the IMD pathway. Nor are they involved in the transcription of major known IMD pathway factors (Fig. 2). However, our results clearly show that they regulate the IMD pathway by targeting factors that are downstream of PGRP-LC/IMD and upstream of Rel2; furthermore, our results indicate that they are likely to act together, since we saw no synergistic effect when the two genes were simultaneously silenced (Fig. 1) and studies in other species have also showed the two proteins act together in regulating gene expression. The two genes could be involved in the transcription of some other unknown IMD pathway factor(s), or function in ways other than regulation of the transcriptional machinery. Recent studies have shown that mediator complexes can also regulate alternative splicing of pre-mature RNAs through the MED23 subunit [36]. Although being components of the mediator complex, Kto and Skd may regulate the IMD pathway through other unknown mechanisms.

The IMD pathway is of vital importance for the mosquitoes’ resistance to *P. falciparum* infection [5], [6], and a better understanding of its regulation may aid in the development of novel malaria control strategies.

## Materials and Methods

### Ethics Statement

This study was carried out in strict accordance with the recommendations in the Guide for the Care and Use of Laboratory Animals of the National Institutes of Health. The protocol was approved by the Animal Care and Use Committee of the Johns Hopkins University (Permit Number: M006H300). Commercially obtained human blood from Interstate Blood Bank Inc was used for parasite cultures and mosquito feeding, and informed consent was therefore not applicable. The Johns Hopkins School of Public Health Ethics Committee approved the protocol.

### Mosquito Rearing

*A. gambiae* Keele strain mosquitoes were maintained on sugar solution at 27°C and 70% humidity with a 12-h light/dark cycle according to standard procedures. Antibiotic treatment of the mosquitoes was performed according to a previous protocol to obtain mosquitoes from which the LB-culturable midgut microbial flora had been eliminated [37].

### Plasmids Construction

The plasmid for over-expression of PGRP-LC1 was constructed previously [33]. To over-express the active forms of Rel2 and IMD, the respective genes were cloned using the following primers: Rel2F, 5′- GCGGCCGCATGTCGACGCTGCTGAATTT-3′; Rel2R, 5′-TCTAGACTTGCGTCCGTCTCCAGCTTGA-3′; IMDF, 5′- GCGGCCGCATGGTGAAGTTTTCAAATTT-3′; IMDR, 5′- TCTAGACTACTACTCCGCTCGGGAGAAT-3′. Each of the four primers was cloned into the pAC5.1/HisV5B vector using the *Not*I/*Xba*I restriction enzyme site. Plasmid DNA was extracted with the Qiagen Endofree kit (Valencia, CA).

### RNAi-Mediated Gene Silencing

Templates for dsRNA (∼500 bp) were prepared by PCR using the following primers: KtoF, 5′-TAATACGACTCACTATAGGGGGCAACGCCGGAATGCCGAAT-3′ and KtoR, 5′-TAATACGACTCACTATAGGGGAACGGCACCCTGATTGACGC-3′; SkdF, 5′-TAATACGACTCACTATAGGGAGTACCTCGCCCACATGAAC-3′ and SkdR, 5′-TAATACGACTCACTATAGGGGAGATCAGCCCGAGAATGAA-3′. The primers for the GFP dsRNA were described previously [8]. The PCR products were purified using a PCR purification kit (Qiagen), and their sequences were confirmed. The dsRNA was generated with an HiScribe T7 In Vitro Transcription Kit (New England BioLabs, Ipswich, MA) according to the manufacturer's instructions. The RNA was ethanol-precipitated and annealed at 65°C in water.

About 69 nl of dsRNA (2–3 µg/µl) in water was introduced into the thorax of cold-anesthetized 2- to 4-day old female mosquitoes using a nano-injector (Nanoject, Drummond) with glass capillary needles according to established methodology. Gene silencing was verified by qRT-PCR.

### qRT-PCR

At 48 h after dsRNA injection, 10 mosquitoes from each replicate were collected and homogenized in lysis buffer. For cell culture, samples were collected 48 h after adding dsRNA. RNA was extracted using the RNeasy kit (Qiagen). Reverse transcription was carried out at 42°C for 2 h using a SuperScript II kit (Invitrogen) and 20-µl reaction mixtures containing oligo(dT) primers and 2 µg of total RNA. qRT-PCR assays were performed according to a standard protocol [12] using SybrGreen PCR Master Mix (Applied Biosystems) and the ABI StepOne real-time PCR system. The relative -fold induction or repression of gene expression in the experimental samples was determined by comparing these values to their respective controls after normalizing the transcript levels with the *A. gambiae* ribosomal S7 gene. The primers used for qRT-PCR are listed in Table S3.

### Transfection

The *A. gambiae* cell line L3–5 [31] was grown in S2 medium (Sigma Aldrich, St. Louis, MO) supplemented with 10% fetal bovine serum (FBS, Invitrogen, San Diego, CA). Approximately 5×10^5^ cells were seeded per well in 24-well plates and maintained until they reached 70%–90% confluency. Transfections were carried out with Effectene (Qiagen). For the over-expression of various genes, expression plasmids were transfected into the cells together with: (i) a plasmid carrying the firefly luciferase gene under the control of the *Cec1* promoter element, and (ii) pRL-Act5C carrying the *Renilla* luciferase (Promega, Madison, WI) gene under the control of the *Drosophila melanogaster Actin 5C* promoter [32]. Cells were then harvested 24 h later for dual luciferase assays. If RNAi-mediated inhibition was used, it was carried out 24 h before transfection. Double-stranded RNA (7 µg per well) was added to the medium without FBS and used to replace the old medium of the confluent cells. Verification of gene silencing was done via qRT-PCR.

### Dual Luciferase Assay

Cells were lysed in a passive lysis buffer at 24 h after the transfection and assayed with the dual luciferase system according to the manufacturer's instructions (Promega). Each experiment was repeated three times, with three independent measurements in each repeat.

### Challenge with Bacteria and *Plasmodium*

Gram-positive (*S. aureus)* and Gram-negative (*E. coli* DH5α) bacteria were cultured in LB broth overnight, washed three times with PBS, and resuspended in PBS. At 4 days after dsRNA treatment, the anesthetized mosquitoes were injected with 69 nl of either *S. aureus* (CFU/mL = 2.5×10^9^) or *E. coli* (CFU/mL = 4.0×10^9^) into the hemolymph, using a microcapillary Nanoject II injector (Drummond). Control dsRNA*-*treated mosquitoes were injected with 69 nl of sterile PBS. Dead mosquitoes were counted and removed daily over an 8-day period. The results shown here were representative of 40–50 mosquitoes for each treatment and at least three independent experiments per tested group. A Kaplan-Meier survival analysis was used to determine the significance of the differences observed.

*P. falciparum* and *P. berghei* infections were administered according to standard protocols [12]. For *P. falciparum* infections, mosquitoes were fed on NK54 gametocytes (provided by the Johns Hopkins Malaria Institute Core Facility) in human blood through a membrane feeder at 37°C 4 days after dsRNA treatment. Unfed mosquitoes were removed within 24 h after feeding, and the rest were maintained at 27°C for 7 days. For *P. berghei* infections, mosquitoes were fed on Swiss-Webster mice infected with the wild-type ANKA strain of *P. berghei* [38] at 21°C 4 days after dsRNA treatment. Unfed mosquitoes were removed from the group within 24 h after feeding, and the rest were maintained at 21°C for 14 days. For both infections, mosquito midguts were dissected. *P.falciparum*-infected midguts were stained with mercurochrome, and oocyst numbers were recorded using a light-contrast microscope (Olympus). *P. berghei* oocyst numbers were directly recorded under a fluorescent microscope (Leica) without staining. Each assay was done with at least 25 mosquitoes, and the data represent the results of three independent assays. P-values were determined using a Mann-Whitney test.

## Supporting Information

Table S1 Survival analysis of control GFP dsRNA- injected mosquitoes compared to Kto dsRNA- or Skd dsRNA- injected mosquitoes after *E. coli* challenge.
(DOCX)

Table S2 Survival analysis of control GFP dsRNA- injected mosquitoes compared to Kto dsRNA- or Skd dsRNA-injected mosquitoes after *S. aureus* challenge.
(DOCX)

Table S3 Primers used for qRT-PCR.
(DOCX)

## References

[1] Meister S, Koutsos AC, Christophides GK (2004) The Plasmodium parasite–a ‘new’ challenge for insect innate immunity. Int J Parasitol 34: 1473–1482. 15582524 10.1016/j.ijpara.2004.10.004

[2] Michel K, Kafatos FC (2005) Mosquito immunity against Plasmodium. Insect Biochem Mol Biol 35: 677–689. 15894185 10.1016/j.ibmb.2005.02.009

[3] Chen Y, Weng ZH, Zheng LB (2008) Innate immunity against malaria parasites in Anopheles gambiae. Insect Science 15: 45–52.

[4] Cirimotich CM, Dong Y, Garver LS, Sim S, Dimopoulos G (2010) Mosquito immune defenses against Plasmodium infection. Dev Comp Immunol 34: 387–395. 20026176 10.1016/j.dci.2009.12.005 PMC3462653

[5] Garver LS, Dong Y, Dimopoulos G (2009) Caspar controls resistance to Plasmodium falciparum in diverse anopheline species. PLoS Pathog 5: e1000335. 19282971 10.1371/journal.ppat.1000335 PMC2647737

[6] Dong Y, Das S, Cirimotich C, Souza-Neto JA, McLean KJ, et al (2011) Engineered anopheles immunity to Plasmodium infection. PLoS Pathog 7: e1002458. 22216006 10.1371/journal.ppat.1002458 PMC3245315

[7] Mitri C, Jacques JC, Thiery I, Riehle MM, Xu J, et al (2009) Fine pathogen discrimination within the APL1 gene family protects Anopheles gambiae against human and rodent malaria species. PLoS Pathog 5: e1000576. 19750215 10.1371/journal.ppat.1000576 PMC2734057

[8] Dong Y, Dimopoulos G (2009) Anopheles fibrinogen-related proteins provide expanded pattern recognition capacity against bacteria and malaria parasites. Journal of Biological Chemistry 284: 9835–9844. 19193639 10.1074/jbc.M807084200 PMC2665105

[9] Riehle MM, Xu J, Lazzaro BP, Rottschaefer SM, Coulibaly B, et al (2008) Anopheles gambiae APL1 is a family of variable LRR proteins required for Rel1-mediated protection from the malaria parasite, Plasmodium berghei. PLoS One 3: e3672. 18989366 10.1371/journal.pone.0003672 PMC2577063

[10] Povelones M, Waterhouse RM, Kafatos FC, Christophides GK (2009) Leucine-rich repeat protein complex activates mosquito complement in defense against Plasmodium parasites. Science 324: 258–261. 19264986 10.1126/science.1171400 PMC2790318

[11] Meister S, Kanzok SM, Zheng XL, Luna C, Li TR, et al (2005) Immune signaling pathways regulating bacterial and malaria parasite infection of the mosquito Anopheles gambiae. Proc Natl Acad Sci U S A 102: 11420–11425. 16076953 10.1073/pnas.0504950102 PMC1183586

[12] Dong Y, Aguilar R, Xi Z, Warr E, Mongin E, et al (2006) Anopheles gambiae immune responses to human and rodent Plasmodium parasite species. PLoS Pathog 2: e52. 16789837 10.1371/journal.ppat.0020052 PMC1475661

[13] Blandin S, Shiao SH, Moita LF, Janse CJ, Waters AP, et al (2004) Complement-like protein TEP1 is a determinant of vectorial capacity in the malaria vector Anopheles gambiae. Cell 116: 661–670. 15006349 10.1016/s0092-8674(04)00173-4

[14] Treisman J (2001) Drosophila homologues of the transcriptional coactivation complex subunits TRAP240 and TRAP230 are required for identical processes in eye-antennal disc development. Development 128: 603–615. 11171343 10.1242/dev.128.4.603

[15] Janody F, Martirosyan Z, Benlali A, Treisman JE (2003) Two subunits of the Drosophila mediator complex act together to control cell affinity. Development 130: 3691–3701. 12835386 10.1242/dev.00607

[16] Rau MJ, Fischer S, Neumann CJ (2006) Zebrafish Trap230/Med12 is required as a coactivator for Sox9-dependent neural crest, cartilage and ear development. Dev Biol 296: 83–93. 16712834 10.1016/j.ydbio.2006.04.437

[17] Hong SK, Haldin CE, Lawson ND, Weinstein BM, Dawid IB, et al (2005) The zebrafish kohtalo/trap230 gene is required for the development of the brain, neural crest, and pronephric kidney. Proc Natl Acad Sci U S A 102: 18473–18478. 16344459 10.1073/pnas.0509457102 PMC1311743

[18] Yoda A, Kouike H, Okano H, Sawa H (2005) Components of the transcriptional Mediator complex are required for asymmetric cell division in C-elegans. Development 132: 1885–1893. 15790964 10.1242/dev.01776

[19] Zhang H, Emmons SW (2000) A C-elegans mediator protein confers regulatory selectivity on lineage-specific expression of a transcription factor gene. Genes & Development 14: 2161–2172. 10970880 10.1101/gad.814700 PMC316889

[20] Wang JC, Walker A, Blackwell TK, Yamamoto KR (2004) The Caenorhabditis elegans ortholog of TRAP240, CeTRAP240/let-19, selectively modulates gene expression and is essential for embryogenesis. Journal of Biological Chemistry 279: 29270–29277. 15073178 10.1074/jbc.M401242200

[21] Clayton JE, van den Heuvel SJL, Saito RM (2008) Transcriptional control of cell-cycle quiescence during C. elegans development. Dev Biol 313: 603–613. 18082681 10.1016/j.ydbio.2007.10.051 PMC2386670

[22] Wang X, Yang N, Uno E, Roeder RG, Guo S (2006) A subunit of the mediator complex regulates vertebrate neuronal development. Proc Natl Acad Sci U S A 103: 17284–17289. 17088561 10.1073/pnas.0605414103 PMC1859923

[23] Carrera I, Janody F, Leeds N, Duveau F, Treisman JE (2008) Pygopus activates Wingless target gene transcription through the mediator complex subunits Med12 and Med13. Proc Natl Acad Sci U S A 105: 6644–6649. 18451032 10.1073/pnas.0709749105 PMC2373359

[24] Moghal N, Sternberg PW (2003) A component of the transcriptional mediator complex inhibits RAS-dependent vulval fate specification in C. elegans. Development 130: 57–69. 12441291 10.1242/dev.00189

[25] Lim J, Lee OK, Hsu YC, Singh A, Choi KW (2007) Drosophila TRAP230/240 are essential coactivators for Atonal in retinal neurogenesis. Dev Biol 308: 322–330. 17585897 10.1016/j.ydbio.2007.05.029 PMC1994652

[26] Conaway RC, Sato S, Tomomori-Sato C, Yao T, Conaway JW (2005) The mammalian Mediator complex and its role in transcriptional regulation. Trends Biochem Sci 30: 250–255. 15896743 10.1016/j.tibs.2005.03.002

[27] Malik S, Roeder RG (2010) The metazoan Mediator co-activator complex as an integrative hub for transcriptional regulation. Nat Rev Genet 11: 761–772. 20940737 10.1038/nrg2901 PMC3217725

[28] Malik S, Roeder RG (2005) Dynamic regulation of pol II transcription by the mammalian Mediator complex. Trends Biochem Sci 30: 256–263. 15896744 10.1016/j.tibs.2005.03.009

[29] Bjorklund S, Gustafsson CM (2005) The yeast Mediator complex and its regulation. Trends Biochem Sci 30: 240–244. 15896741 10.1016/j.tibs.2005.03.008

[30] Bourbon HM (2008) Comparative genomics supports a deep evolutionary origin for the large, four-module transcriptional mediator complex. Nucleic Acids Res 36: 3993–4008. 18515835 10.1093/nar/gkn349 PMC2475620

[31] Muller HM, Dimopoulos G, Blass C, Kafatos FC (1999) A hemocyte-like cell line established from the malaria vector Anopheles gambiae expresses six prophenoloxidase genes. Journal of Biological Chemistry 274: 11727–11735. 10206988 10.1074/jbc.274.17.11727

[32] Zheng XL, Zheng AL (2002) Genomic organization and regulation of three cecropin genes in Anopheles gambiae. Insect Mol Biol 11: 517–525. 12421409 10.1046/j.1365-2583.2002.00360.x

[33] Chen Y, Ling EJ, Weng ZH (2009) Functional characterization of PGRP-LC1 of Anopheles gambiae through deletion and RNA interference. Insect Science 16: 443–453.

[34] Lin H, Zhang LM, Luna C, Hoa NT, Zheng LB (2007) A splice variant of PGRP-LC required for expression of antimicrobial peptides in Anopheles gambiae. Insect Science 14: 185–192.

[35] Cirimotich CM, Ramirez JL, Dimopoulos G (2011) Native microbiota shape insect vector competence for human pathogens. Cell Host Microbe 10: 307–310. 22018231 10.1016/j.chom.2011.09.006 PMC3462649

[36] Huang Y, Li W, Yao X, Lin QJ, Yin JW, et al (2012) Mediator Complex Regulates Alternative mRNA Processing via the MED23 Subunit. Mol Cell 45: 459–469. 22264826 10.1016/j.molcel.2011.12.022 PMC3288850

[37] Antonova Y, Alvarez KS, Kim YJ, Kokoza V, Raikhel AS (2009) The role of NF-kappaB factor REL2 in the Aedes aegypti immune response. Insect Biochem Mol Biol 39: 303–314. 19552893 10.1016/j.ibmb.2009.01.007 PMC2702699

[38] Franke-Fayard B, Trueman H, Ramesar J, Mendoza J, van der Keur M, et al (2004) A Plasmodium berghei reference line that constitutively expresses GFP at a high level throughout the complete life cycle. Mol Biochem Parasitol 137: 23–33. 15279948 10.1016/j.molbiopara.2004.04.007

