# Supplementary material for: Transcriptional Mediators Kto and Skd Are Involved in the Regulation of the IMD Pathway and Anti-Plasmodium Defense in Anopheles gambiae
Source: PLoS One. 2012 Sep 25;7(9):e45580. doi: 10.1371/journal.pone.0045580 (PMC3458077; doi:10.1371/journal.pone.0045580)
Supplement: Table S1 — Survival analysis of control GFP dsRNA- injected mosquitoes compared to Kto dsRNA- or Skd dsRNA- injected mosquitoes after E. coli challenge. (DOCX) [file pone.0045580.s001.docx]

**Table S1.** Survival analysis of control GFP dsRNA- injected mosquitoes compared to Kto dsRNA- or Skd dsRNA- injected mosquitoes after *E. coli* challenge.

|  | dsGFP | dsKto | | dsSkd | |
| --- | --- | --- | --- | --- | --- |
| Exp. | N | N | *p* | N | *p* |
| #1 | 50 | 50 | 0.0403 | 50 | 0.0207 |
| #2 | 40 | 40 | 0.017 | 40 | 0.0048 |
| #3 | 40 | 40 | 0.0317 | 40 | 0.0228 |
