# Supplementary material for: Transcriptional Mediators Kto and Skd Are Involved in the Regulation of the IMD Pathway and Anti-Plasmodium Defense in Anopheles gambiae
Source: PLoS One. 2012 Sep 25;7(9):e45580. doi: 10.1371/journal.pone.0045580 (PMC3458077; doi:10.1371/journal.pone.0045580)
Supplement: Table S2 — Survival analysis of control GFP dsRNA- injected mosquitoes compared to Kto dsRNA- or Skd dsRNA-injected mosquitoes after S. aureus challenge. (DOCX) [file pone.0045580.s002.docx]

**Table S2.** Survival analysis of control GFP dsRNA- injected mosquitoes compared to Kto dsRNA- or Skd dsRNA-injected mosquitoes after *S. aureus* challenge.

|  | dsGFP | dsKto | | dsSkd | |
| --- | --- | --- | --- | --- | --- |
| Exp. | N | N | *p* | N | *p* |
| #1 | 50 | 50 | 0.0265 | 50 | 0.0039 |
| #2 | 40 | 40 | 0.0253 | 40 | 0.0133 |
| #3 | 40 | 40 | 0.0447 | 40 | 0.0127 |
