# Supplementary material for: Transcriptional Mediators Kto and Skd Are Involved in the Regulation of the IMD Pathway and Anti-Plasmodium Defense in Anopheles gambiae
Source: PLoS One. 2012 Sep 25;7(9):e45580. doi: 10.1371/journal.pone.0045580 (PMC3458077; doi:10.1371/journal.pone.0045580)
Supplement: Table S3 — Primers used for qRT-PCR. (DOCX) [file pone.0045580.s003.docx]

**Table S3.** Primers used for qRT-PCR.

| Gene | Forward (5`-3`) | Reverse (5`-3`) |
| --- | --- | --- |
| *Kto* | CGTAACGATGGGCAACATGG | GCTGCTGATTCTGGGGCATA |
| *Skd* | AGCTGGATTTGGGAGAGGAT | GCATGTTGATCTGCTGCATT |
| *RPS7* | CCATCCTGGAGGATCTGGTA | GATGGTGGTCTGCTGGTTCT |
| *Imd* | GAATTTCCCAAATGGTGTG | TGTGTAGATTGCTCGCGTTC |
| *Dredd* | CGGCGTGGAGAGTAATGTTT | TTCGAGCGATGACGTTACTG |
| *Fadd* | CTGGCACTGGACACAAAAGA | TTCCAGCTTTTGCCAATTTC |
| *Ikk-γ* | TCTGTCCAAGCACATCGAAC | CACTTGTTCCGCTGTTTTCA |
| *Tak1* | GGAGTTGCAGCTCAAGAAGG | GCTGCCGATTCTTATCGTTC |
| *Rel2* | CGGAGAAGTCGAAGAAAACG | CACAGGCACACCTGATTGAG |
| *Fbn9* | CGCTCCCTGTTCGAGCTGCA | TTGTGGTACGTCAGCGAGT |
